# Effects of Nutrition Education on Improving Knowledge and Practice of Complementary Feeding of Mothers with 6- to 23-Month-Old Children in Daycare Centers in Hawassa Town, Southern Ethiopia: An Institution-Based Randomized Control Trial

**DOI:** 10.1155/2020/6571583

**Published:** 2020-08-24

**Authors:** Selam Deksiyous Muluye, Tefera Belachew Lemma, Tona Zema Diddana

**Affiliations:** ^1^School of Nutrition, Food Science and Technology, Hawassa University, Hawassa, Ethiopia; ^2^College of Public Health and Medical Sciences, Jimma University, Jimma, Ethiopia

## Abstract

Undernutrition and hidden hunger threaten the survival, growth, and development of children, young people, economies, and nations. Inappropriate complementary feeding practice due to poor maternal knowledge and awareness in combination with low income and infectious disease is the contributing factor for child undernutrition. Hence, this study was aimed at determining the effect of nutrition education on improving the knowledge and practice of complementary feeding of the mothers with 6- to 23-month old children in daycare centers of Hawassa Town, Southern Ethiopia. An institution-based randomized control trial design was employed. Daycare centers were randomly allocated for the intervention group and the control group. Among the total daycare centers in the town, five were assigned to receive nutrition education and the rest five for the control group (CG). The simple random sampling technique used to select individual participants from each daycare center. Two hundred (200) mother-child pairs (100 for each group) were recruited. Sociodemographic and economic variables were collected by the structured questionnaire. Knowledge of appropriate complementary feeding was assessed by seven knowledge questions. Appropriate complementary feeding practice was assessed by adapting Alive and Thrive Infant and Young Child Feeding (IYCF) practice guidelines. Nutrition education was given for four consecutive months by using Alive and Thrive IYCF guidelines. Data were analyzed by the SPSS software program version 20. The chi-squared test was used to test the significant differences in the proportion of good knowledge and good practice of complementary feeding and good dietary diversity between two groups. The independent *t* test was used to test the significant differences in mean dietary diversity between two groups. At 95% confidence interval, *p* < 0.05 was considered statistically significant. The results revealed that the proportion of mothers with good knowledge of appropriate complementary feeding was increased from 59% at pretest to 96% at posttest and the appropriate complementary feeding practice was improved from 54% at pretest to 86% at posttest in IG. There was no change in the knowledge and practice of complementary feeding practice in CG after four months. The proportion of mothers with good complementary knowledge was 54% both at pretest and at posttest and good complementary feeding practice was 51% and 52% at pre- and posttest in CG, respectively. There was no significant difference (*p* > 0.05) on complementary feeding knowledge and practice between two groups at pretest, while the difference was highly significant (*p* < 0.05) at the posttest. In conclusion, providing nutrition education improved the appropriate complementary feeding knowledge and practice of mothers. In recommendation, government and other partners working on sustainable child nutrition reduction should focus on the nutrition education to improve the knowledge and appropriate complementary feeding practice including daycare centers.

## 1. Background

Undernutrition and hidden hunger threaten the survival, growth, and development of children, young people, economies, and nations [1]. The period from birth to two years is believed to be a window of opportunity for not only avoiding undernutrition but also its long-term adverse consequences. Poor nutrition during this critical period will have a greater risk of dying, illnesses, such as diarrhea and respiratory infections, and deficits in cognitive development and school performance [2]. Research evidence suggested that improved complementary feeding ranked to be the third effective preventive actions for reducing under-five mortality and is estimated to have the potential to prevent 6% of all deaths [3].

The age of 6–23 months is the window of opportunity and the important stage to optimize child growth and development to prevent undernutrition: wasting, underweight and stunting, and negative consequences in adulthood [4, 5]. Despite the period is critical and several efforts are made to tackle the problem, childhood undernutrition is still alarmingly high. According to the UNICEF/WHO/World Bank joint report, nearly 149 million under-five children were short for their age (stunted) and more than 49.5 million were wasted. In regions, at least one in every five children was stunted. Africa and Asia bear the greatest share of all forms of malnutrition. In Africa, the magnitude of stunting and wasting was 38% and 28%, respectively [6].

Ethiopia is one of the developing countries with a high prevalence of childhood undernutrition. It was documented that about two out of every five (38.4 percent) children under five years were stunted in Ethiopia [6, 7]. The 2019 Ethiopia Mini Demographic and Health Survey indicated that 37%, 7%, and 21% of under-five children were stunted, wasted, and underweight at the national level, respectively. Additionally, undernutrition is still widely distributed among under-five children with a wide variation among regions in Ethiopia. The magnitude of stunting (13.6%), wasting (2.3%), and underweight (2.4%) was lowest in Addis Ababa. The highest prevalence of stunting, wasting, and underweight was 48.7% in the Tigray region, 21.1% in the Somali region, and 31.7% in the Afar region of Ethiopia. The problem was 36.3% (stunting), 19.8% (underweight), and 6.3% (wasting) in the region where this study was conducted [8]. A longitudinal study conducted in Sothern Ethiopia showed that the prevalence of stunting, underweight, and wasting among children during lent fasting and nonfasting period was 31.6–33.7%, 11.7–15.7%, and 4.4–4.8%, respectively [9]. Another study conducted in the drought-prone area of Ethiopia showed that stunting, wasting, and underweight was 49.4%, 13.7%, and 37.1%, respectively [10].

These high burdens of undernutrition show that little has been achieved by the reduction of childhood undernutrition despite different efforts were undertaken. Chronic undernutrition particularly too short for age increases the risk of mortality and morbidity and poor cognitive and physical development [11, 12], lower economic productivity, and adverse maternal reproductive outcomes in adulthood [13]. Undernutrition among children results from the interactions of multilevel factors such as inadequate dietary intake, repeated and chronic infections, and compromised prenatal conditions [14]. Based on UNICEF 1998 conceptual framework, poor maternal-child feeding practice is the underlying cause of child malnutrition [15]. This poor feeding practice might be due to lack of knowledge and awareness, religious trends, and culture which might contribute significantly to enhance the childhood malnutrition. For instance, a study conducted in the Sidama region of Southern Ethiopia indicated that the nutritional status and feeding practices of 6- to 23-month-old children are affected by maternal fasting during the fasting period. The author described that a small proportion of 6- to 23-month old children, 2.3% during fasting and 6.7% during the nonfasting period, met the minimum acceptable diet [16].

Nutrition is one of the foundations of human health and development. Infant and young child feeding practice comes among the most effective intervention to improve child health and nutrition. It also recognized that investing in nutrition including child feeding is also economically sound and has been identified as a “best” investment [17]. This critical investment saves mothers' and children's lives and improves children's education outcomes, which, in turn, boosts the economic productivity. The government of Ethiopia planned the “Seqota” Declaration is a special commitment to reduce child malnutrition by 2030 in collaboration with other sectors such as the Ministry of Agriculture and Natural Resources (MOANR), Ministry of Livestock and Fishery Resource Development (MOLF), and Ministry of Health (MOH). One of the key goals of this declaration is to achieve zero stunting in children less than 2 years by 2030. Besides, Ethiopia is currently updated and implementing its National Nutrition Program II (NNP-II) with a special focus on the first 1,000 days in strategic objectives [18]. For achieving the goal of undernutrition reduction, children should take appropriate complementary food in addition to breast milk. However, the government of Ethiopia is implementing projects and programs, and existing evidence from the local and national studies showed that child feeding practice is suboptimal. The data from EDHS 2016 also indicated that child feeding practice was suboptimal at the national level. For instance, only 67% of Ethiopian children had age-appropriate breastfeeding, 60% introduce complementary feeding at age of 6 months, 14% meet minimum dietary diversity, 45% get recommended meal frequency, and only 7% met minimum acceptable diet [7]. Another study from a local survey in Southern Ethiopia indicated that appropriate feeding among infants and the young child was 9.5% [19].

Recommendation by different scholars indicated that nutritional education and counseling are needed to enhance knowledge [19, 20] and awareness on complementary feeding practice of the mothers/caregivers in the communities and promotion of context-specific child feeding practices [21]. On the other hand, the existing nutrition education researches were focused on a community level and other institutions but did not give any attention to daycare centers. Therefore, this study was aimed to assess the effects of nutrition education on improving the knowledge and practice of complementary feeding of mothers with 6- to 23-month-old children in daycare institutions in Hawassa City, Southern Ethiopia, 2018 G.C.

## 2. Description of the Study Area

The intervention was conducted in Hawassa town, the capital of the south nation, nationalities, and peoples' regional state (SNNPR). The town located 273 km far from Addis Ababa, the capital of Ethiopia. It has an area of 157.21 square kilometers, 8 subcities, and 32 kebeles. The town experiences a moderate type of tropical climate. It is situated in its relatively high altitude about 1800 m above sea level, the average annual rainfall is 1000 mm (lowest 800 mm and highest 1300 mm), and an average temperature of 22°C. Based on city administration, there are 10 daycare centers and 292 children (6–24 months) in Hawassa City (source: Hawassa City Administration Health Bureau, 2016).

## 3. Study Design and Study Period

A single-blind institution-based randomized control trial with pretest and posttest design was used from March to June 2018 G.C.

## 4. Source Population and Study Population

All mothers of children 6–24 months in daycare institutions of Hawassa City were the source population. Mothers of children aged 6–20 months in daycare institutions during the baseline data collection were selected for this study as study population.

## 5. Inclusion and Exclusion Criteria

Children in daycare institutions within complementary food feeding age were considered as inclusion criteria. The exclusion criteria were as follows: mothers with identified mental illness during baseline data collection period and children with medically restricted to take any food items. However, there were no participants who met the exclusion criteria.

## 6. Sample Size Determination

The sample size (*n*) was determined based on the difference between two population proportions. The proportion of appropriate child complementary feeding practice (P2 = 62.9%) was used from the previous study [22] for the control group. Since there was no such research conducted in the area, the authors expected to improve the proportion of appropriate complementary feeding practice by 30% after nutrition education in the intervention group. Accordingly, hypothesizing expected change of 30% in this study, the proportion of appropriate complementary feeding practice (P1 = 30% + 62.9% = 92.9%) was used for the intervention group. The level of confidence (*α*) at 95% confidence interval was taken to be 0.05 (Z*α*/2 = 1.96) and the power (1 − *β*) 100% was taken to be 80% (Z2 = 0.84). Ten percent (10%) of calculated sample size was addded to compensate loss to follo-up and minimize attrition bias. The intracluster correlation coefficient (ICC) was 0.11 and −0.18 for complementary feeding knowledge and appropriate complementary feeding practice, respectively, suggesting that clustering was safe to ignore for the study population. Hence, using the following formula (equation (1)), the total final sample size included in this study was found to be 200 (100 for each group) 6- to 20-month-old children:

(1) $$\begin{array}{c} n=\frac{{\left(Z_{1}+Z_{2}\right)}^{2}*2P\left(1-P\right)}{{\left(P_{2}-P_{1}\right)}^{2}}. \end{array}$$

## 7. Sampling Procedures and Technique

**Figure 1:**
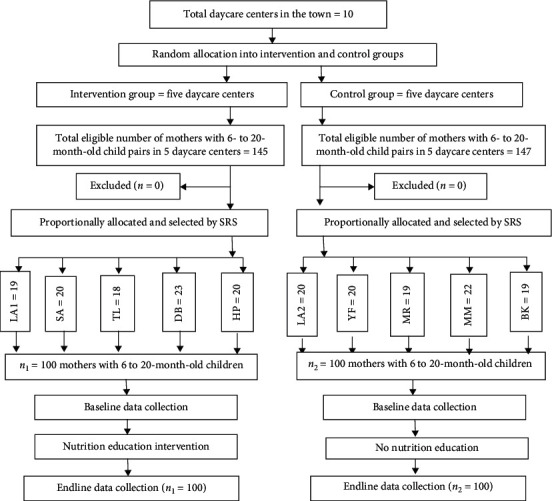
Schematic diagram for sampling techniques and group assignment. LA1 = little angles 1 (*N* = 26), SA = S. Arsema (*N* = 28), TL = true love (*N* = 28), DB = dibora (*N* = 32), HP = hope (*N* = 31), LA2 = little angles 2 (*N* = 28), YF = Yenat Fikir (*N* = 28) MR = Mercy (*N* = 29), MM = Mami (*N* = 32), BK = Barkot (*N* = 30), SRS = simple random sampling.

The sampling procedures and techniques of study participants are shown in Figure 1. Two steps were followed to assign and select participants. First, the random allocation of daycare centers into the intervention group and the control group was made. Five-day care centers were randomly assigned for the intervention group, and the other five daycare centers were assigned for the control group in a ratio of 1 : 1. Secondly, the lists of all children between 6 and 24 months old were identified in each daycare center. Lists of 6- to 24-month-old children (sampling frame) were made. Finally, the required sample size was selected using a simple random sampling technique. Random numbers were generated using ENA-for-SMART software after.

## 8. Data Collection Instruments and Procedures

The Ethiopian Demographic and Health Survey (EDHS) 2016 [7] questionnaire was adapted to collect the sociodemographic and economic variables. Pretest and posttest mothers/caregivers knowledge and complementary feeding practice were assessed by adapting EDHS 2016 infant and young child feeding (IYCF) questionnaires [7] and Alive and Thrive Ethiopia Indicator Guide [23]. Seven complementary feeding knowledge questions were designed to assess the knowledge of mothers on complementary feeding. Participants were given score 1 if they correctly answer the knowledge question, and score 0, if they did not correctly answer the question. Then, the correct score was summed to create a knowledge score. Similarly, complementary feeding practice was assessed using 12 appropriate feeding practices. The score was given one (1) for correct and favorable or healthy complementary feeding practice and Zero (0) for not wrong answer and not favorable or unhealthy complementary feeding practices. A score of <50% was classified as poor while that of 50% and more was classified as good for either complementary feeding knowledge or complementary feeding practice [24].

The pretest and posttest minimum dietary diversity was determined with reference to the World Health Organization (WHO) recommendations. Seven food groups were listed, and the mothers were interviewed to respond weather the child consumed or not the specified food groups within the past 24 hours prior to data collection. Each of the 7 food groups was allocated a score of 1 if a child consumed a specified food group or zero (0) if not consumed. The dietary diversity scores were recorded as good if a child consumed four or more food groups or low if a child ate less than four food groups [25, 26].

## 9. Nutrition Education Intervention

Nutrition education was designed based on the evidence of complementary feeding practice gaps in the community and baseline survey of this study. After identifying the existing knowledge and practice of complementary feeding, the study objective was set. Then, the nutrition education of mothers was started immediately after baseline data were collected. Nutrition education was provided in Amharic, the local language, by trained nutritionists. Education was given for four consecutive months on a biweekly basis. The duration of education was 2 hours per session. Face-to-face education was provided in addition to the practical demonstration. Active lecturer, posters, note pad, brochures, and practical demonstration sessions were used during nutrition education. Alive and Thrive manual standard-based posters and guidelines were used for the session of nutritional education [23]. Key messages included in education were complementary food recipes and preparation, appropriate amount and frequency of feeding, personal hygiene and sanitation, and dietary diversification. Practical demonstration of complementary food preparation, dietary diversification, recipes preparation, and personal hygiene and sanitation practices was intensively done. During these sessions, the standard checklist was prepared using alive and thrive complementary feeding guideline to confirm that the protocol was followed. Demonstration and redemonstration by the active participation of participants were conducted. At the posttest after imparting nutrition education for 4 months, the same questionnaire was used to evaluate their knowledge and change in their complementary feeding practice. After data collection, the same message that the intervention group received during nutritional education was given for three hours to the control group for ethical purposes.

## 10. Data Quality Control

The questionnaire first prepared in English was translated into the local language (Amharic). The questioner was pretested on 5% of the total sample size to check validity and clearness in the area other than this study was conducted. Six nutrition graduates were trained in appropriate questionnaire administration techniques, objectives of the study, and consent taking techniques prior to data collection. The response to each questionnaire was checked for completeness and consistency before leaving the field. One health extension worker and two nutrition professionals experienced in research and nutrition education or IYCF practice were selected and received two days of training on all procedures of nutrition education. Posttest data were collected using the same semistructured questionnaire as the pretest. The questioner was administered by the same trained data collectors to avoid personal intervariability [27].

## 11. Variables

### 11.1. Dependent Variables

  Knowledge of complementary feeding
  Practices of complementary feeding

### 11.2. Independent Variables

Sociodemographic and economic characteristic and previous exposure of the mothers to the nutrition information were the independent variables.

### 11.3. Data Processing and Analysis

Pearson's chi-squared test was used to see the difference in sociodemographic and economic variables between the two groups. The normality of data was checked by using the Kolmogorov–Smirnov test. Variance inflation factors (VIFs) were used to check multicollinearity. The difference in the proportion of mothers' knowledge and practice towards appropriate complementary feeding and dietary diversity was assessed by the chi-squared test at both pretest and posttest. The independent Student's *t* test was used to check the statistically significant difference in mean dietary diversity score between two groups. At 95% confidence, variables with probability value (*p* value) less than 0.05 were considered as statistically significant. Finally, the result was summarized using frequency, mean, standard deviation, and percentage.

### 11.4. Ethical Consideration

Ethical clearance was obtained from Institutional Review Board of Hawassa University. Written consent was obtained from the study participants. Information provided by the participants was held strictly confidential.

## 12. Result

### 12.1. Sociodemographic and Economic Characteristics of the Participants

The sociodemographic and economic characteristics of the participants are indicated in Table 1. The chi-squared test indicated that there was a significant difference (*p* < 0.05) in respondents' age, occupation, household head, number of under-five children and child age between two groups. However, then there was no significance difference on the rest of sociodemographic variables.

### 12.2. Knowledge of Mother on Complementary Feeding

The knowledge of the complementary feeding of the mother is described in Table 2. The proportion of mothers with good knowledge was increased from 59% (*n* = 59) at pretest to 96% (*n* = 96) at posttest in the intervention group (IG), while it was 54% (*n* = 54) at both pretest and posttest in the control group (CG). Pearson's chi-squared test showed that there was no significant difference (*p*=0.954) in complementary feeding knowledge between two groups at pretest while the difference was statistically significant (*p*=0.027) at the posttest.

### 12.3. Practices of Mothers on Complementary Feeding

The complementary feeding practice of mothers of 6- to 23-month-old children is indicated in Table 3. Seventy-four percent (*n* = 74) of mothers in IG and 48% (*n* = 48) in CG prefer porridge for complementary food at pretest, while 97% (*n* = 97) in IG and 51% (*n* = 51) in CG prefer porridge for complementary food at posttest. The proportion of mothers washing hands during childcare increased from 88% at baseline to 100% at the end of the intervention in IG. The proportion of mothers mixing pulses and cereals based on a recommendation (pulses: cereals 1 : 3) was increased from 16% at baseline to 91% at the endline in IG. On the other hand, the cereal and pulse processing practice (soaking and dehulling) was improved in the intervention group. The consumption of animal source food (meat, egg, milk, and products) and vitamin A-rich fruits and vegetables slightly improved after intervention in IG. For instance, meat/fish/poultry, egg, and dairy products improved from 16%, 20%, and 66% at baseline to 36%, 81%, and 32% at the endline. The aggregate complementary feeding practice indicated that proportion of mothers following good complementary practice changed from 54% (*n* = 54) at pretest to 86% (*n* = 86) at posttest in IG while the pretest and posttest good complementary feeding practice was 51% (*n* = 51) and 52% (*n* = 52) in CG, respectively. There was no significant difference (*p*=0.308) on the proportion of mothers practicing appropriate (good complementary feeding) at pretest between two groups by the chi-squared test. On the contrary, there was a significance difference (*p* < 0.01) between two groups at the end of the study.

### 12.4. Dietary Diversity of Infants of Complementary Feeding Age

The child dietary diversity score (DDS) is indicated in Table 4. The result revealed that the proportion of children who had eaten at least four food groups among seven (good dietary diversity) was increased at pretest to posttest (26–67%) in IG. The proportion of children who consumed recommended (at least four food groups) DDS was 23% and 33% at pretest and posttest in CG, respectively. The independent *t* test showed that there was no significant difference (*p*=0.811) in mean dietary diversity at pretest while the difference was highly significant (*p* < 0.01) at the posttest. Specifically, intake of nutrient-dense animal source food improved in IG as described in Table 3.

## 13. Discussion

Childhood undernutrition and feeding practice is a major concern in Ethiopia. Poor dietary intake, poor maternal and childcare practice and poor hygiene and sanitation practice due to lack of knowledge and awareness of mothers/caregivers on inappropriate infant and young child feeding practice in combination with culture and belief are contributing factors. Optimal complementary feeding depends on accurate information and skilled support from the family, community, and health system. Hence, the aim of this study was to determine the effect of nutrition education on improving knowledge and practices of mothers on complementary feeding of 6- to 23-month-old children in daycare centers at Hawassa town, Southern Ethiopia. The result revealed that the proportion of good knowledge of mothers on complementary feeding was increased from 59 to 96% in the IG. There was no significant change in the knowledge level of mothers in the control group. The proportion of mothers practicing appropriate complementary feeding was improved (54–86%) to 70% at the end of the study in IG. There was no change in pretest and posttest appropriate complementary feeding practice (51–52%) in the control group.

The effectiveness of nutrition education on improving knowledge and practice of complementary feeding was in agreement with the finding from Kenya such that the mean nutrition knowledge was significantly higher in IG than that of the control group at the end of the study [22]. The finding is also comparable to another study from China. The author revealed that a one-year educational intervention showed significant change in mother's knowledge of complementary feeding practices in IG, and the difference between the two groups was statistically significant [28]. Another study from Southern Ethiopia indicated that nutrition education intervention of mother with 6- to 23-month-old children significantly improves the knowledge, attitude, and practice of mother on pulse use in complementary food [29]. Likewise, cluster-randomized trial on complementary and responsive feeding education to caregivers in Indians found that complementary feeding messages delivered through home visits were effective in changing the knowledge and behaviors among mothers/caregivers in both IG at 6 and 12 months of the intervention [30]. Similarly, a study from Southern Ethiopia showed that both study groups were similar at baseline for knowledge and practices of complementary feeding, but there was a significant improvement only in the IG [31]. The finding of this study is also in agreement with the study from Indonesia [32], Uganda [33], Kenya [34], and Pakistan [35].

Even though there was a significant improvement in nutritional knowledge (96%), nearly 14% did not practice good complementary feeding practices. This might be because maternal-infant and young child feeding practice could be compromised by sociodemographic and economic factors such as educational status and occupation of household [36], household food security status [37], household income [38], and the number of under-five children [19].

In this study, proportion of mothers, incorporating animal source foods and vitamin-rich fruit and vegetables, increased at the end of education. Accordingly, change in consumption of meat/fish/poultry, egg, vitamin-rich fruits and vegetables, and dairy products were 16–36%, 20–32%, 15–30%, and 66–81% in IG, respectively. This finding is in line with a study finding from rural China such that significantly higher proportion of mothers/caregivers in the IG used different food items including meats, eggs, beans, dark green leafy vegetables, and fruits than those in the control group to feed their children at the end of the nutrition education intervention [39]. Even though there was a significant improvement in the inclusion of different food groups in both studies, there was variation proportion. The variation in two studies might be explained by the difference in length of the nutrition education period since long-term exposure to information may have more power to influence the behavior of participants. On the other hand, the practice of complementary feeding is always influenced by other factors especially food availability at home, attitude of the caregiver, and family socioeconomic status as well as mothers who prepare food for the family. Consequently, the difference might also be due to these factors.

In this study, the proportion of children who met the minimum dietary diversity (consumed at least four food groups) was increased (26–67%) in IG. After four months of nutrition education intervention, one additional food group was incorporated into complementary food on average in IG. The finding is comparable with that of the study from a cluster-randomized control trial study conducted in Uganda where the child DDS was improved by 6 folds at 3 months compared with that at baseline in IG [33]. The finding is also in agreement with a study from Ethiopia where six months of nutrition education of mothers/caregivers improved the dietary diversity of young children [31]. The finding is also in agreement with the study documented from Peru such that there is a significant improvement in infant and young child dietary diversity because of 18 months of nutrition education intervention [40]. The significant improvement of knowledge and practice of complementary feeding of mothers as well as the inclusion of food groups and dietary diversity might be due to exposure to nutrition information, routine teaching, and demonstration. The improvement might also be explained by the improvement of mothers' complementary feeding knowledge during nutrition education intervention. This suggests that nutrition education had the power to change the existing poor nutritional knowledge and awareness concomitantly improving complementary feeding practice and dietary diversity of infants and young children. The slight (10%) increment in dietary diversity in the control group might be due to women's follow-up of growth monitoring and promotion program and postnatal care follow-up for vaccination because when they attend the health facilities, the likelihood of getting counseling is high.

The limitation of this study is that it did not collect household food security status both pretest and posttest since it is one of the underlying causes of childhood malnutrition and can compromise maternal-child feeding practice.

## 14. Conclusion

Providing nutrition education improved the appropriate complementary feeding knowledge practice of mothers in daycare centers.

## 15. Recommendations

Government and other partners working on sustainable child nutrition reduction should focus on nutrition education to improve the knowledge and appropriate complementary feeding practice including daycare centers.

## Figures and Tables

**Table 1:** Sociodemographic and economic characteristics of mothers with children (6–20 months old) in the daycare center at Hawassa City, Southern Ethiopia, 2018 G.C . (*n*_1_ = *n*_2_ = 100).

Variable	Allocation group		*X* ^2^ (df), *p* value
	IG, *n* (%)	CG, *n* (%)	
Respondent age, in years			
18–24	42 (42)	32 (32)	8.970 (2), 0.011^*∗*^
25–35	50 (50)	62 (62)	
>35	8 (8)	6 (6)	

Religion			
Protestant	52 (52)	44 (44)	6.186 (3), 0.103
Orthodox	31 (31)	32 (32)	
Muslim	10 (10)	21 (21)	
Catholic	7 (7)	3 (3)	

Marital status			
Married	94 (94)	90 (90)	1.230 (2), 0.541
Divorced	3 (3)	4 (4)	
Widowed	3 (3)	6 (6)	

Occupation			
Employed	38 (38)	24 (24)	26.40 (4),<0.001
Merchant	33 (33)	16 (16)	
Student	2 (2)	19 (19)	
Housewife	27 (27)	41 (41)	

Head of the household			—
Male-headed	27 (37.0)	46 (63.0)	7.802 (2), 0.020^*∗*^
Female-headed	21 (58.3)	15 (41.7)	
Jointly	52 (57.1)	39 (42.9)	

Number of children <5 year			
≤2 children	92 (92.0)	96 (96.0)	4.308 (1), 0.038^*∗*^
>2 children	8 (8.0)	4 (4.0)	

Average monthly of income (ETB)			
≤5000	53 (53.0)	69 (69.0)	12.659 (1),<0.001
>5000	47 (47.0)	31 (31.0)	

Age of the child in completed months			
≤12 months	34 (34.0)	30 (30.0)	4.367 (1), 0.037^*∗*^
12–20 months	66 (66.0)	70 (70.0)	

Sex of the child			
Female	54 (54.0)	59 (59.0)	0.020 (1), 0.887
Male	46 (46.0)	41 (41.0)	

IG = intervention group; CG = control group.

**Table 2:** Knowledge of mothers on complementary feeding for children (6–20 months old) in daycare institutions at Hawassa City, Southern Ethiopia, 2018.

Knowledge variables	Study period	Study group		*p* value^*∗*^
		IG, *n* (%)	CG, *n* (%)	
Know the time to start complementary food	Baseline	94 (94)	91 (91)	
	Endline	100 (100)	95 (95)	0.812

Know the nutritive benefit of complementary food	Baseline	74 (74)	69 (69)	0.138
	Endline	100 (100)	66 (66)	<0.001^*∗*^

Know what should be used in the preparation of complementary foods	Baseline	43 (43)	55 (55)	0.205
	Endline	99 (99)	59 (59)	<0.001^*∗*^

Know the amount of complementary food to feed the child	Baseline	34 (34)	26 (26)	0.171
	Endline	92 (99)	34 (34)	<0.001^*∗*^

Know the frequency of food to feed the child	Baseline	34 (34)	31 (31)	0.221
	Endline	98 (98)	40 (40)	<0.001^*∗*^

Type of complementary food	Baseline	55 (55)	55 (55)	0.221
	Endline	95 (95)	61 (61)	<0.001^*∗*^

Thickness/consistency of complementary foods	Baseline	38 (38)	41 (41)	0.053
	Endline	87 (87)	55 (55)	<0.001^*∗*^
Aggregate complementary feeding knowledge^¶^				

Good	Baseline	59 (59)	54 (54)	=0.954
	Endline	96 (96)	54 (54)	<0.01)

IG = intervention group; CG = control group, ^*∗*^the difference is significant at a probability value of 5%, ¶ = the remaining proportion belongs to poor complementary feeding knowledge, $ = *p* value for the chi-squared test.

**Table 3:** Practice of mothers on complementary feeding for children (6–20 months) in daycare institutions at Hawassa City, Southern Ethiopia, 2018.

Variable	Allocation group			
	Baseline		Endline	
	IG, *n* (%)	CG, *n* (%)	IG, *n* (%)	CG, *n* (%)
Types of food commonly prepared by you				
Gruel	14 (14)	46 (46)	2 (1)	46 (46)
Porridge	74 (74)	48 (48)	97 (97)	51 (51)
Bread (kita)	12 (12)	7 (7)	1 (1)	6 (6)

Hand wash when caring the child				
No	12 (12)	45 (45)	0 (0)	45 (45)
Yes	88 (88)	55 (55)	100 (100)	55 (55)

Proportions of pulses to cereals you are mixing				
1/4^th^ pulse and 3/4^th^cereals	16 (16)	19 (19)	91 (91)	20 (20)
1/2 pulse and 1/2 cereals	35 (35)	49 (49)	6 (6)	49 (49)
3/4^th^pulse and 1/4^th^cereals	49 (49)	32 (32)	3 (3)	31 (31)

Remove (dehulling) the outer cover				
No	0 (.0)	0 (0)	80 (80)	9 (9)
Yes	100 (100)	100 (100)	20 (20)	91 (91)

Soaking and/or germination				
No	95 (11.0)	99 (99)	49 (5)	84 (84)
Yes	5 (5.0)	1 (1)	51 (95)	16 (16)

Cereals/roots and tubers				
Yes	62 (62)	75 (75)	97 (92.0)	90 (90)

Pulses and nuts				
Yes	41 (41)	53 (53)	83 (94.0)	62 (62)

Dairy and products				
Yes	66 (66)	46 (46)	81 (81)	54 (54)

Eggs				
Yes	20 (20)	27 (27)	32 (32)	24 (24)

Meat, fish, and poultry				
Yes	16 (16)	8 (8)	36 (36)	21 (21)

Vitamin A-rich vegetables and fruits				
Yes	15 (15)	18 (18)	30 (30)	21 (21)

Other fruits and vegetables				
Yes	35 (35)	33 (33)	44 (44)	35 (35)

*Complementary food feeding practice* ^*∗*^			*pvalue* ^*$*^	
Good (baseline)	54 (54)	51 (51)	0.308	
Good (endline)	86 (86)	52 (52)	<0.01	

IG = intervention group; CG = control group, ^*∗*^the remaining proportion belongs to poor complementary feeding practice, $ = *p* value for the chi-squared test.

**Table 4:** Dietary diversity score status of children (6–23 months old) in daycare institutions at Hawassa City, Southern Ethiopia, 2018.

Dietary diversity score	Study period	Intervention group	Control group	*p* value^*∗*^
Good, (%)	Pretest	26	23	0.519
	Posttest	67	33	0.034

Mean (SD)	Pretest	2.55 (1.60)	2.60 (1.34)	0.811
	Posttest	4.03 (1.36)	3.07 (1.03)	<0.01

SD = standard deviation; ^*∗*^the value is from the chi-squared test for % of good dietary diversity and independent *t* test for mean dietary diversity score.

## Data Availability

The datasets used and analyzed for this study are available from the corresponding author upon reasonable request.
